# Existence of Gender-Based Difference in Morphology of Convex Lateral Tibial Plateau in Korean Population Primary Knee Joint Osteoarthritis

**DOI:** 10.1155/2021/6641717

**Published:** 2021-08-25

**Authors:** Ji-Hoon Nam, Yong-Gon Koh, Paul Shinil Kim, Kyoung-Tak Kang

**Affiliations:** ^1^Department of Mechanical Engineering, Yonsei University, 50 Yonsei-ro, Seodaemun-gu, Seoul 03722, Republic of Korea; ^2^Joint Reconstruction Center, Department of Orthopaedic Surgery, Yonsei Sarang Hospital, 10 Hyoryeong-ro, Seocho-gu, Seoul 06698, Republic of Korea; ^3^Department of Orthopaedic Surgery, The Bone Hospital, 67, Dongjak-daero, Dongjak-gu, Seoul, Republic of Korea

## Abstract

**Purpose:**

Morphological differences in the knee joints of females and males have been reported in a previous study. These differences have realized the need of developing a gender-specific prosthesis. However, anatomical studies on gender-based differences in the proximal tibial plateau's sagittal curvature have rarely been conducted. Therefore, this study is aimed at evaluating the geometry of the sagittal curvature of the proximal tibial plateau in the Korean population.

**Methods:**

Three-dimensional data for the sagittal curvature of the tibial plateau morphology from 1976 patients (i.e., 299 male and 1677 female) were assessed using magnetic resonance imaging. The sagittal profiles of the tibial plateaus were also evaluated. The independent *t*-test and paired *t*-test were used for statistical analysis.

**Results:**

The proximal tibia had concave and convex surfaces in the medial and lateral plateaus, respectively, for both genders. In addition, the medial diameter of the tibial plateau was significantly greater than the lateral diameter for both genders. Gender-based difference was not found in the medial diameter of the tibial plateau but was observed in the lateral diameter.

**Conclusion:**

These results may provide guidelines for a suitable knee implant design for the Korean patients. The incorporation of this shape information in the medial and lateral sides in the prosthetics for a total knee arthroplasty and a lateral unicompartmental knee arthroplasty can improve knee range motion.

## 1. Introduction

In recent studies, considerable attention has been devoted to gender-based differences of total knee arthroplasty (TKA). A new implant design was introduced to reflect the gender differences [1, 2]. In the last few decades, the knee joint has been widely investigated in the morphologic view, and shape differences have been found between ethnicity and gender [3]. Western female and male subjects have been characterized as having well-known geometric variations [4–6]. However, quantitative information on the geometric differences between knee joints in female and male subjects of Asian–Pacific origin are relatively lacking. In addition, such studies only focus on the anteroposterior (AP) to mediolateral (ML) dimensions for male and female knees. Yue et al. recently evaluated the sagittal profiles of the distal femoral condyle and the proximal tibial plateau [7]; however, the size of samples in their study was very low.

The design of the tibial articular surfaces in TKA has a significant impact on the knee joint function. In arthroplasty that preserves bilateral or posterior cruciate ligaments, movement restrictions occur when the conformity between femur and tibial articular surface is high [8]. In contrast, if the tibial surfaces are of low conformity, excessive anterior–posterior excursion of the femoral–tibial contact points may cause tibial component rocking in some cases and possible loosening [8]. In fact, the medial and lateral tibial plateaus are quite different.

The major difference between the lateral and medial tibial plateaus is slightly concave and convex tibial plateaus. The medial ligaments are much tighter and stronger than the lateral. In addition, the dynamic stability controlled by the iliotibial band and popliteus is important. During knee flexion, the medial condyle of femur moves posteriorly. But the movement is less than lateral condyle. The lateral condyle moves quite a bit posteriorly during flexion [9]. Even with the flat tibial plateau of unicompartmental knee arthroplasty, this posterior displacement of natural knee could not be achieved [10]. Therefore, kinematics of the normal knee could be implemented by a convex lateral tibial component [10]. No study on the sagittal curvature of the tibial plateau in the Asian population has yet been published for a large number of samples. Compared to the Western population, the Asian population has a higher prevalence of knee osteoarthritis [11], and the frequency of TKAs in the Asia–Pacific region is increasing [12]. Considering this, the gender-based differences of knee morphometry must be quantitatively investigated for the Asia–Pacific population.

Therefore, this study is aimed at first evaluating the diameter of the medial and lateral tibial plateaus in the sagittal plane and then determining whether gender-based differences exist. We hypothesize herein that gender-based differences exist in the sagittal diameters of the medial and lateral tibial plateaus.

## 2. Materials and Methods

**Figure 1:**
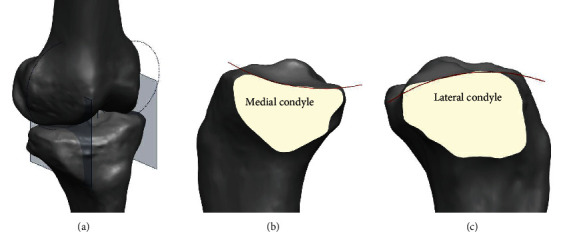
Schematic representation of tibia sagittal (a) coordination, (b) medial condyle, and (c) lateral condyle.

A total of 2155 OA knees (i.e., 310 in the male subjects and 1845 in the female subjects) were investigated herein. Each patient agreed this study, and the approval of the institute of review board was obtained by author's hospital. The patients with primary knee OA and who underwent TKA were only included. Patients who have any history of other surgery or trauma on the knee were excluded. Finally, 1976 patients comprising 1677 females and 299 males were included in this study. The female subjects had an average age of 68.9 ± 6.6 years, while the male subjects had an average age of 69.2 ± 6.6 years. The average body mass index (BMI) of the female subjects was 30.8 ± 3.4 kg/m^2^, while that of the male subjects was 29.6 ± 3.2 kg/m^2^. Magnetic resonance imaging (MRI) scans of each patients were acquired. The magnetic field strength of MRI scanner (Achieva 1.5 T; Philips Healthcare, Best, Netherlands) was 1.5 T. The sagittal plane slice thickness was 1 mm to obtain a high-resolution image for the tibiofemoral knee joint. The axial plane slice thickness was 5 mm for the hip and the ankle joints. To make the condition nonfat saturated, an axial proton-density sequence was contained. A resolution was set as high option for the spectral presaturation inversion recovery sequence (repetition time: 3590.8 ms; echo time: 25.0 ms; number of excitations: 2.0; acquisition matrix: 512 × 512 pixels; field of view: 140 × 140 mm) [13]. The MRI scan files were imported to a segmentation software (Mimics version 17.0; Materialise, Leuven, Belgium). By segmenting each slice of MRI, 3D models of tibial bone and cartilage were constructed. The femur and the tibia were aligned using both mechanical axes. The tibial rotation was defined using PCL foot print to medial 1/3 tuberosity landmarks. We defined the sagittal plane for the medial and lateral tibial plateaus using the femoral coronal circular center as the sagittal location (Figure 1(a)). The outer contour of the condyle in the medial and lateral tibial sagittal planes was defined as a circle (Figures 1(b) and 1(c)). The diameter of each circle was then determined.

All measurements were performed by 5 years of experienced observer. The intraobserver and interobserver variabilities were tested using 3D MRI scans from 100 female and male patients. After the primary measurements by the same observer, each were remeasured by a second observer. The interval between two measurements was more than 1 week. The intraobserver reliability was 0.89, while the interobserver reliability was 0.93.

## 3. Statistical Analysis

Statistical analyses were performed using R (version 4.0.3; R Core Team). The independent *t*-test was used to calculate the *p* value which decide the significant differences in data between both genders in 1976 patients. The statistics used for the analysis of the descriptive data of sample were mean and standard deviation. In addition, paired *t*-tests were used for the difference within the same gender. *p* = 0.05 was used as significant level. The power analysis was performed using G power 3.1. The statistical power was 99.9%, and the alpha value was 0.05. The measured data that used as a input parameter of power analysis was lateral diameter of the female and male. The intra- and interexaminer reliability was evaluated using the intraclass correlation method.

## 4. Results

There were no significant differences among the demographics for each group, including BMI and age (Table 1). The proximal tibia for both genders had concave and convex surfaces in the lateral and medial plateaus. The mean diameters of the lateral and medial tibial plateaus were 113.8 ± 80.1 mm and 76.1 ± 36.9 mm, respectively. The medial tibial plateau diameter for both genders was significantly greater than the lateral tibial plateau diameter. No gender-related difference was found in the medial tibial plateau diameter; however, a gender-based difference was found in the lateral tibial plateau diameter (Table 2).

## 5. Discussion

The key finding of this study is that a gender-based difference exists in the lateral diameter of tibial plateau. The medial tibial plateau diameter was significantly greater than the lateral tibial plateau diameter. However, no gender-based difference exists in the medial tibial plateau diameter. Therefore, our hypothesis is partially accepted.

Using 3D computational models of the proximal tibial plateau, this study showed that the diameter of medial side was significantly greater than that of the lateral in both genders. This tendency was also found in previous studies [7]. Yue et al. showed that the medial and lateral diameters of tibial plateaus were 121.8 ± 9.6 mm and 102.0 ± 13.8 mm, respectively, in female [3, 7]. For male, the diameters of the medial and lateral tibial plateaus were 132.2 ± 8.6 mm and 120.4 ± 12.8 mm, respectively [7]. Asseln et al. showed that the lateral tibial plateau diameter was smaller than the medial tibial plateau diameter [3].

Yue et al. found that the tibial plateau diameter in male was significantly greater than that in female. They showed that the differences disappeared by the normalization of the radii using the anteroposterior lengths of the lateral and medial plateaus. The data showed that the gender-based differences in the proximal tibial curvature may not need to be considered in the tibial component design of a TKA. This study showed that the lateral tibial plateau diameter in male was significantly greater than that in female; however, no statistical difference was found in the medial tibial plateau diameter. These findings could be explained by the different cohorts and demonstrate the structural changes of the proximal tibial plateau profile because of osteoarthritis. Wahl et al. found a gender-based difference in the lateral tibial plateau diameter, supporting our result [14]. This tendency was also found in our result of the female lateral tibial plateau being more convex.

As previously mentioned, the design of the tibial insert surfaces in TKR has important consequences to the joint function [8]. However, the tibial insert surfaces in widely used TKA have been designed to be dependent to the femoral component. In other words, the convex anatomy characteristics in the lateral tibial plateau were disregarded. Varadarajan et al. recently studied the kinematics of cruciate-retaining (CR) total knee implant with the anatomic articular surface. They compared the kinematics between anatomic and contemporary CR implants applying various loading conditions [15]. Their study showed that across different loading of various activities, the anatomic CR showed kinematic patterns of normal knee than those of contemporary CR implants. Particularly, for deep knee bend and chair sit activities, a medial pivot motion is shown only in the anatomic CR. The other contemporary CR implants showed lateral pivot or no pivot motion and paradoxical anterior femoral motion [15]. Koh et al. recently investigated the preservation of normal knee biomechanics using specific articular surface conformity in a customized posterior stabilized- (PS-) TKA using a computational simulation [16]. The results indicated that the TKR design with the anatomical geometry provided a better normal-like biomechanical effect compared to the TKR design with the tibia insert surface dependent on the femoral component even though they are different, customized TKAs [16]. They also recently conducted wear performance evaluation using a computational simulation to compare three customized PS-TRKA which have different conformities of the tibiofemoral articular surface [17]. The results showed that the changes in the conformity of tibiofemoral articular surface result in different kinematics and contact mechanics during a gait cycle [17]. Although an anatomic mimetic design TKA could not show the best wear performance, it showed a good performance in kinematics compared to other designs. In addition, the enhancement in the kinematical and mechanical performance can be achieved through the optimization of tibiofemoral articular surface conformity [17]. The aforementioned study showed how important the preservation of the convex characteristics in the lateral tibial plateau is in the TKA performance [15–17]

Such a lateral tibial plateau design can be applied to the lateral UKA design. The isolated arthritis in the lateral side of the knee is not common, and the treatment is difficult; hence, it much less often occurs compared to the medial side [18]. The best treatment of isolated arthritis is UKA. UKA maintains normal anatomy and executes the kinematics of normal knee. However, the outcomes of the lateral UKA are unsatisfactory and have not been as good as those of the medial UKA [19]. Majority of the previous studies showed that 10-year survival rate is approximately 80% [20]. In the knee, the lateral compartment has fundamental different with respect to the medial compartment, which may explain that the medial UKA compartment are not as much suitable for the lateral. Accordingly, it may be reasonable to use different components in the lateral compartment. The most important difference between the medial and lateral compartments is that the lateral tibia has convex plateau, whereas the medial is slightly concave. In low knee flexion, the plateau was contact with femur in larger radius. On the contrary, in high flexion, the plateau was contact with femur in smaller radius [10]. If the tibial plateau is flat, the median changes in the flexion gap were 2 mm until 130° flexion and start to be large beyond 130° flexion. The average range in the lateral UKR is 110°, it is acceptable to use flat plateau [21]. However, the range of motion increase up to 130° in the medial compartment with a minimally invasive surgery [22]. Therefore, the previous study expected that the average range of motion of lateral UKR with a minimally invasive will also reach to approximately 130° or could be more [10]. These phenomena could cause the kinematic problems in a flat tibial plateau. In terms of clinical relevance, our result showed that the lateral tibial plateau diameter was different from the medial tibial plateau diameter. Our results have thus far provided a basis, but should be carefully used. Furthermore, our result demonstrated that the tibial plateau morphology should be considered as patient-specific rather than gender-specific manner. Even in the new TKA designed to provide high flexion, the failure of achieving full flexion often occurs. If they do, they encounter problems. In the normal knee, lateral femoral condyle subluxes to the posterior direction relative to tibia in high flexion. But current TKA design do not allow this. If the convex tibial plateau and movable bearing are applied in the lateral TKA, the high flexion could be achieved. This study has several limitations. First, MRI was used to create a 3D model of the tibia, which may lead to errors than using CT. Nevertheless, soft tissues, such as the articular cartilage can be reconstructed using MRI. The inaccuracy in the 3D model constructed form MRI could be minimized using the protocol described in a previous study [23]. Second, this study did not report postoperative clinical outcomes because we did not investigate patients with UKA implants. Third, this study has sample size difference between male and females. But it implies clinical situation that the majorities of the patients are female. Nevertheless, valuable parametric information for designing TKA and UKA, which are well-matched to the anatomy of Korean patients, was provided in the findings of this study.

## 6. Conclusions

The present study highlighted the finding of a gender-based difference in the lateral tibial plateau diameter, which was more convex in female. The results may provide guidelines for a knee implant design suitable for the Korean population. The incorporation of this shape in the medial and lateral sides of a TKA and a lateral UKA can improve flexion.

## Figures and Tables

**Table 1:** Comparison of the age and BMI between Korean males and females.

Parameter	Whole patients (*n* = 1976)	Female (*n* = 1677)	Male (*n* = 299)	*p* value
	Mean ± SD	Mean ± SD	Mean ± SD	
Age	69.0 ± 6.8	68.9 ± 6.6	69.2 ± 6.6	n.s
BMI (kg/m^2^)	30.8 ± 3.4	31.0 ± 3.4	29.9 ± 3.5	n.s

n.s: nonsignificant; BMI: body max index; SD: standard deviation.

**Table 2:** Comparison of tibial sagittal diameter between Korean males and females.

Parameter	Whole patients (*n* = 1976)	Female (*n* = 1677)	Male (*n* = 299)	*p* value
	Mean ± SD (range)	Mean ± SD (range)	Mean ± SD (range)	
Medial diameter (mm)	113.8 ± 80.1 (17.8, 1050.2)	113.9 ± 81.0 (17.8, 1050.2)	112.9 ± 75.0 (17.9, 456.4)	n.s
Lateral diameter (mm)	76.1 ± 36.9 (19.6, 456.0)	74 ± 35.9 (19.6, 439.3)	87.6 ± 40.3 (33.8, 456.0)	<0.01
	<0.01	<0.01	<0.01	

n.s: nonsignificant.

## Data Availability

The data are available from the corresponding author upon reasonable request.
